# On the Use of Cod Liver Oil in Nursing Sore Mouth

**Published:** 1851-11

**Authors:** 


*On the Use of Cod Liver Oil in Nursing Sore Mouth.* By JOHN EVANS, M. D., Prof. of Obstetrics, &c., in Rush Medical College.

The extensive prevalence in the west, of a form of disease in women, generally attending the period of lactation, which has in consequence acquired the name of "Nursing Sore Mouth," and the general want of satisfactory success in its treatment, have induced me to give the result of my observations upon its nature and management.

The disease generally affects females of delicate constitution and spare habit, in which the function of assimilation is but imperfectly performed. It not unfrequently makes its appearance in such during the last months of gestation, but much oftener during the period of lactation.

The diagnostic symptoms are a burning sensation in the mouth, as if it had been scalded, which is greatly aggravated by hot drinks, attended at first by but little redness, and followed by small ulcerations upon the tongue and different parts of the buccal cavity. In some cases, instead of these ulcers, there is a diffused redness of the mucous membrane of the mouth. These symptoms are generally attended and often preceded by a burning sensation in the stomach, pyrosis, indigestion, and occasionally vomiting. The bowels are most frequently relaxed, and in some cases an obstinate diarrhœa attends.

The course of the disease is often variable, sometimes for a few days being almost entirely relieved and again recurring. As has been observed by Prof. Brainard, it is often attended by ulcerations in the vagina and upon the mucous surfaces of the labia, which generally grow worse as the irritation of the mouth subsides, and vice versa. The wasting of the system often continues, if the child is kept at the breast, without the function of nutrition being improved by regimen or treatment, until the patient sinks and dies of marasmus and its attendant local lesions.

Nursing Sore Mouth is a disease of debility, consequent upon the marasmus produced by imperfect nutrition and the demand upon the system of gestation and lactation, and generally speedily gets well after weaning the child, unless it has continued so long as seriously to have impaired the function of nutrition. Profuse hæmorrhages and copious lochial discharges, favor its development.

Treatment by a resort to medication, especially mercurial, generally aggravates rather than relieves the disease. Although in some instances symptoms may be temporarily palliated by the use of the bitter tonics, and astringents such as nitrate of silver, tannin, &c., I have thought in the end they do more harm than good, as there are few, if any, of this class of remedies that do not, under the circumstances, ultimately act as irritants. The ulcers in the mouth may generally be promptly, but temporarily relieved, by the application to each of a little pure muriatic acid, applied by dipping a small point of a feather or a pencil in the acid and touching it to the ulcerated surfaces. Although they speedily heal after this application, others soon make their appearance, unless the general condition of the system is relieved.

In some instances, after having failed to relieve either the diarrhœa or irritation of the mouth by the ordinary means of treating these symptoms in other cases, I have observed a marked improvement by abandoning medication altogether, and placing the patient upon an animal diet and the free use of mucilaginous drinks.

Observing the influence of Cod Liver oil in preventing the wasting of the tissues of the body in cases of marasmus, especially from phthisis and tabes mesenterica, it occurred to me that its influence might be equally beneficial in the disease in question. The diarrhœa and ulcerations of the mucous surfaces being in many cases similar to those produced by the marasmus in those affections. I have accordingly been in the habit of prescribing it taken in French brandy or malt liquor, as might be found best suited to the taste, or most convenient, and generally with the happiest effects. Where the patient can be induced to continue its free use, it has uniformly proved beneficial, and in most instances effected a cure. If treatment should fail to relieve the disease, a resort to weaning the child should never be deferred until the patient loses her strength so that she cannot maintain the erect position. — *Ibid.*

